# Multidetector CT of expected findings and complications after hysterectomy

**DOI:** 10.1007/s13244-018-0610-9

**Published:** 2018-04-06

**Authors:** Massimo Tonolini

**Affiliations:** Department of Radiology, “Luigi Sacco” University Hospital, Via G.B. Grassi 74, 20157 Milan, Italy

**Keywords:** Hysterectomy, Laparoscopic surgery, Haemorrhage, Ureter, X-ray computed tomography

## Abstract

**Abstract:**

Indicated to manage a variety of disorders affecting the female genital tract, hysterectomy represents the second most common gynaecological operation after caesarean section. Performed via an open, laparoscopic or vaginal approach, hysterectomy is associated with non-negligible morbidity and occasional mortality. Iatrogenic complications represent a growing concern for gynaecologists and may result in prolonged hospitalisation, need for interventional procedures or repeated surgery, renal impairment and malpractice claims. As a result, radiologists are increasingly requested to investigate patients with suspected complications after hysterectomy. In the vast majority of early postoperative situations, multidetector CT represents the ideal modality to comprehensively visualise the surgically altered pelvic anatomy and to consistently triage the varied spectrum of possible injuries. This pictorial review provides an overview of current indications and surgical techniques, illustrates the expected CT appearances after recent hysterectomy, the clinical and imaging features of specific complications such as lymphoceles, surgical site infections, haemorrhages, urinary tract lesions and fistulas, bowel injury and obstruction. Our aim is to increase radiologists’ familiarity with normal post-hysterectomy findings and with post-surgical complications, which is crucial for an appropriate choice between conservative, interventional and surgical management.

**Teaching points:**

• *Hysterectomy via open, laparoscopic or vaginal route is associated with non-negligible morbidity.*

*• Multiplanar CT imaging optimally visualises the surgically altered pelvic anatomy.*

*• Familiarity with early post-hysterectomy CT and expected findings is warranted.*

*• Complications encompass surgical site infections, haemorrhages, bowel injury and obstruction.*

*• Urological complications include ureteral leakage, bladder injury, urinomas and urinary fistulas.*

## Introduction

### Background

Performed to manage several different disorders of the female reproductive system, hysterectomy (surgical removal of the uterus) represents one of the most prevalent surgeries worldwide and the second most common gynaecological operation after caesarean section. The annual hysterectomy rates vary among different countries in the range 1.2 to 4.8/1000 women. In the USA, approximately 600,000 such operations are performed each year [1].

Although the majority of women experience an uneventful postoperative course, hysterectomy is associated with non-negligible risks. Potentially serious postoperative complications such as infections, haemorrhage, urinary and bowel injuries may be either recognised intraoperatively, manifest during postoperative hospitalisation, or sometimes become apparent weeks or months later [2–5].

### Aim

Transvaginal ultrasound represents the first-line modality to investigate the female genital organs. However, in recently operated patients this modality is cumbersome and poorly tolerated, and provides a limited field of view. In our experience, partly due to fear of litigation, radiologists are increasingly requested to investigate suspected iatrogenic complications after hysterectomy. During the last decade, multidetector CT has become the mainstay technique to assess the vast majority of postoperative abdominal and pelvic conditions, as it rapidly and comprehensively visualises the surgically altered anatomy and can triage the varied spectrum of possible complications [6–9].

This pictorial essay provides an overview of current indications and surgical techniques, then reviews and illustrates the expected postoperative CT findings, and common and unusual specific complications after hysterectomy. Our aim is to provide radiologists with an increased familiarity in the interpretation of early post-hysterectomy CT studies, thus providing a consistent basis for correct therapeutic choice and ultimately helping to decrease morbidity [7, 9].

## Overview of hysterectomy

### Techniques and indications

The uterus may be removed using three different routes, namely vaginal, abdominal and laparoscopic. The indications of different surgical techniques are summarised in Table 1.

**Table 1:** Surgical techniques for hysterectomy with relative indications and contraindications

Route	Indications	Contraindications
Vaginal hysterectomy (VH)	Genital prolapse (50–65% of cases) Hypermenorrhoea/dysfunctional uterine bleeding Symptomatic (bleeding) uterine leiomyomas Microinvasive cervical carcinoma	History of caesarean section (CS) or other pelvic surgery No previous vaginal delivery Large uterus (≥12–14-week gestation size) Coexistent extrauterine pelvic pathology (e.g. adhesions, endometriosis) Need for oophorectomy Invasive tumours
Laparoscopically assisted VH (LAVH)	Dysfunctional uterine bleeding or symptomatic uterine leiomyomas in patients with contraindicated or difficult VH (e.g. due to previous CS or adhesions) Patients with chronic pelvic inflammatory disease (PID) requiring hysterectomy Patients with endometriosis requiring hysterectomy	Obesity Very large uterus Potentially malignant adnexal mass Risk of laparotomic conversion (e.g. severe post-surgical adhesions, endometriosis requiring bowel resection and/or involving rectovaginal septum)
Total laparoscopic hysterectomy (TLH)	Same as LAVH + endometrial and cervical tumours	
Abdominal hysterectomy (AH)	Malignant genital tumours Potentially malignant adnexal mass Uterine leiomyomas not amenable to VH and laparoscopy (e.g. very large uterus, severe adhesions) Endometriosis and PID not amenable to laparoscopy (e.g. due to rectovaginal septum involvement, need for bowel resection) Secondary post-partum haemorrhage (exceptional)	Benign uterine disease (e.g. dysfunctional uterine bleeding or symptomatic uterine leiomyomas) amenable to VH or laparoscopy

Although declining, abdominal hysterectomy (AH) continues to be the most widely used approach (60–64% of all operations) worldwide. AH begins with a transverse suprapubic laparotomic incision of the anterior abdominal wall, involves dissection and removal of the uterus, and ends with suture of the vaginal cuff (VC). Radical AH for tumours is completed with removal of the adnexa (salpingo-oophorectomy), lymphadenectomy, peritoneal exploration and lavage [10, 11].

Worldwide, the majority (90%) of hysterectomies are performed to treat non-neoplastic conditions which negatively impact women’s quality of life, such as bleeding leiomyomas and hypermenorrhoea. In recent years, hysterectomy rates are declining in Western countries after introduction of several alternative therapeutic options for benign diseases, such as hormonal medications, intrauterine levonorgestrel-releasing devices, endometrial ablation techniques, hysteroscopic and surgical myomectomy, ultrasound focused energy and transcatheter uterine artery embolisation. However, leiomyomas still account for approximately 40–62% of all hysterectomies [1, 12–15].

For benign disorders requiring hysterectomy, according to the American College of Obstetricians and Gynaecologists guidelines, criteria for choosing the surgical route include surgeon’s skill and training, parity, size and shape of the vagina and uterus, accessibility to the uterus (e.g. prolapse, pelvic adhesions), underlying pathological condition and presence of extrauterine disease [14].

In vaginal hysterectomy (VH) the uterus is approached, excised, anteverted and extracted via the vagina. Being the safest technique, unless contraindicated, VH should be the preferred approach in the majority of patients with non-malignant disorders, particularly genital prolapse [12–14, 16].

Laparoscopy is increasingly used as a minimally invasive alternative to AH for those patients in whom VH is contraindicated or technically challenging, and currently accounts for 14% of all hysterectomies in the USA. The technically challenging total laparoscopic hysterectomy (TLH) is differentiated from laparoscopically-assisted VH (LAVH), which is completed with removal of the uterus via the vagina. Compared to AH, both VH and laparoscopy result in shorter hospitalisation, less postoperative pain and fever, and faster return to normal activities. On the other hand, laparoscopy requires specific surgeons’ training and the longest operating time of all techniques [1, 2, 12, 15].

### Complications

The overall morbidity and mortality rates associated with different hysterectomy techniques are summarised in Table 2. Both prevalence of major complications at 30 postoperative days and mortality are highest after radical hysterectomy for cancer. Conversely, the composite morbidity is much lower in VH compared to AH, and in patients operated with every technique for benign disease, the 40-day mortality is approximately 1/1000. General risk factors for post-hysterectomy morbidity included advanced age, medical comorbidities and malignancy [3].

**Table 2:** Overall morbidity, reoperation and mortality rates associated with different hysterectomy techniques

Route	30-day major complications (%)	Need for repeated surgery (%)	Mortality (%)
Radical abdominal hysterectomy (AH) for cancer	9.8	3.0	1.1
AH for benign conditions	4.8	1.9	0.2
Vaginal hysterectomy (VH)	2.4	1.0	0.03
Laparoscopically-assisted VH (LAVH)	3.4	1.5	0.1
Total laparoscopic hysterectomy (TLH)	2.6	2.0	–

Regarding the type of complications, systemic and cardiorespiratory events such as venous thromboembolism, myocardial infarction, pneumonia, sepsis and fluid/metabolic imbalance may develop after hysterectomy similarly to other surgeries. Clinically manifest thromboembolic disease and pneumonia are reported in 1–4% and 2% of patients, respectively [2–5, 12].

Patterns and incidence of specific complications partly differ between the three surgical approaches (Table 3). The commonest category is postoperative infections, which affect 9–13% of patients after hysterectomy with every technique and encompass surgical site, urinary tract, systemic and wound infections. The latter occur in 1–6% of patients, particularly after AH. Vaginal vault haematoma and lymphocele are typically associated with VH and radical AH, respectively. The incidence of significant bleeding requiring transfusions is in the range 2–5% with every technique. The next most frequent category is urologic injuries, which occur most usually after TLH (3–6.2%), LAVH (3.06%) and radical AH (2.78%). The uncommon VC dehiscence mostly develops after TLH (1.35%) rather than LAVH, AH and VH in descending order of frequency. With every technique, potentially severe injuries to the gastrointestinal tract occur in less than 1% (roughly 0.2–0.3%) of patients [2–5, 12].

**Table 3:** Relative frequency of specific post-hysterectomy complications with different surgical techniques

Route	All infections	Wound infections	Lymphocele	Vaginal vault haematoma	Bleeding requiring transfusion	Urologic complications (bladder, ureter, fistulas)	Vaginal cuff dehiscence	Gastrointestinal complications
Abdominal hysterectomy (AH) (* radical AH for malignancy)	+++	++ (* +++)	++ (* +++)	+	++	+/++ (* +++)	+	+
Vaginal hysterectomy (VH)	+++	+	+/−	+++	+	+	+/−	+
Laparoscopically assisted VH (LAVH) Total laparoscopic hysterectomy (TLH)	++	+	+	+	++	+++	++/+++	+

## Early post-hysterectomy CT

### Indications and techniques

In our experience, the usual indications for obtaining urgent post-hysterectomy CT imaging are listed in Table 4, and are often multiple in each patient.

**Table 4:** Usual clinical indications for post-hysterectomy CT imaging

Intraoperative injury (suspected, recognised by surgeon or repaired) to blood vessels, bowel or urinary tract
Suspected haemorrhage: - dropping haemoglobin - blood from drainage tube - hypotension/shock
Physical signs of peritonitis
Persistent or worsening abdominal distension, pelvic or perineal pain
Suspected infection/sepsis: - fever - increasing leukocyte count and C-reactive protein levels
Abnormal vaginal examination: - discharge - suspicious physical finding (e.g. swelling, vaginal cuff discontinuity)
Suspicious, abnormal or extensive transvaginal ultrasound findings (e.g. vaginal vault haematoma or abscess)
Suspected urologic injury: - hydronephrosis - worsening renal function tests - haematuria, abnormal urinalysis

Since pleuropulmonary changes (such as atelectasis, pneumonia and pleural effusions) are commonly encountered, after recent hysterectomy the CT acquisition should encompass the entire abdomen from the lung bases to the inferior contour of the gluteus muscles. A comprehensive CT study including intravenous contrast medium (CM) injection is warranted unless strongly contraindicated. In patients with impaired renal function or allergy, appropriate measures are recommended such as intravenous hydration or pharmacological premedication, following guidelines from the European Society of Urogenital Radiology [17].

Following recent hysterectomy, we generally recommend a multiphase protocol, including a preliminary unenhanced acquisition, aiming to identify high attenuation values consistent with recent blood at the vaginal vault, in the pelvis and peritoneal cavity. After automated power injection of 110–130 ml of iodinated CM (dose may be calculated on the basis of lean body weight and CM iodine concentration) such as iopromide (370 mgI/ml), iomeprol (350 mgI/ml) or iodixanol (300 mgI/ml) at a 2.5 to 4-ml/s flow rate, we usually acquire arterial phase scanning using a bolus tracking technique with a region of interest in the infrarenal aorta, a 10-s delay and a 120-Hounsfield units (HU) threshold. Aimed at detecting active haemorrhage, the arterial phase (CT angiography) may be obviated to limit the radiation dose if haemoglobin levels tend to improved during the first postoperative days. The mandatory portal-venous phase acquisition is obtained after 75–80 s after start of CM injection. At the attending radiologist’s discretion, excretory phase may be acquired after injection of 200 ml of saline and 8–10 min of delay, to visualise the opacified urinary cavities. Additional very delayed acquisition (20 min to 1 h) may be warranted if urinary tract injury or urinoma are suspected [7, 9].

Additionally, in patients with clinical suspicion of iatrogenic bladder injury or fistulisation multidetector CT cystography acquired after drop infusion of 1:10 diluted CM through the Foley catheter represents the most accurate technique. The resulting acquisition should be reviewed along multiple planes at CT angiography window settings (width 600–900 HU, level 150–300 HU) [18]. Borrowing from experience with multidetector CT imaging of acute gynaecological diseases, also in the postoperative setting, routine reconstruction of 3–4-mm-thick contiguous images along sagittal and coronal planes is beneficial to elucidate the female pelvic anatomy [19–21].

### Study interpretation and expected post-surgical appearances

**Fig. 1:**
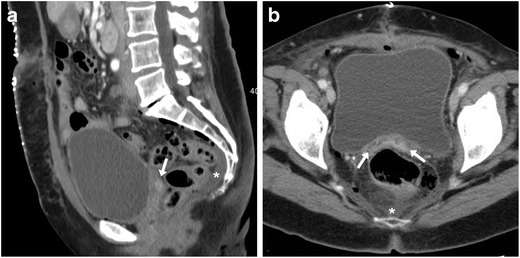
**A**, **B**) Usual contrast-enhanced CT appearance after recent abdominal hysterectomy (AH), including a non-distended vagina (arrows) with slight upwards retraction of the vaginal dome in sagittal view (**A**), preserved band-like transverse configuration and thin mucosal enhancement (**B**), minimal presacral fluid. (Partially reproduced from open access ref. no [58])

After AH the vagina is best identified on mid-sagittal CT images, with the VC slightly retracted cranially compared to preoperative studies, a band- or H-shaped axial configuration, uniform walls and thin enhancing mucosa (Fig. 1). After recent hysterectomy, minimal pelvic effusion and gas are common postoperative findings. Following AH, air may either distribute freely in the peritoneal cavity or collect in the operated subperitoneal spaces, and typically resolves within a week but decreases on serial imaging. Conversely, air which persists or increases should raise concern for visceral perforation. Following laparoscopic surgery, the amount of expected intraperitoneal free gas is lower compared to AH because insufflated carbon dioxide is rapidly reabsorbed, and subcutaneous emphysema from insufflation in the abdominal wall is sometimes encountered [22–24].

**Fig. 2:**
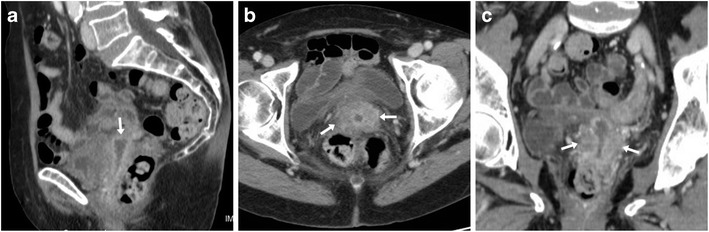
Expected CT appearance 4 days after uncomplicated vaginal hysterectomy (VH) performed to treat genital prolapse (GP), in an 80-year-old female suffering from abdominal distension, vague pelvic pain and low-grade fever. Multiplanar contrast-enhanced images (A–C) show a moderately distended vagina (arrows) with loss of the usual transverse configuration, circumferential mural thickening and marked, uniform mucosal enhancement reflecting diffuse vaginal oedema. The patient was discharged without any additional treatment (Partially reproduced from open access ref. no [58])

After uncomplicated VH, we have consistently observed a characteristic CT appearance which reflects diffuse vaginal oedema, including a roundish configuration in transverse planes, circumferential oedematous mural thickening and marked mucosal enhancement (Fig. 2). When interpreting early post-hysterectomy CT studies, radiologists should thoroughly search for abnormal (bloody, fluid or abscess) collections at the surgical site above the closed VC.

## CT imaging of post-hysterectomy complications

### Vaginal vault haematoma

A typical sequel of VH, vaginal vault haematoma develops in up to 20–25% of patients, more frequently in premenopausal women with larger, more vascular uteri rather than in elderly woman with atrophic uteri. Diagnosed up to 7–14 days after VH, haematomas often manifest with fever, and are generally self-limiting but may require blood transfusions, prolongation of hospitalisation or readmission after discharge. Conversely, after AH and TLH, vaginal vault haematoma is rare and not associated with febrile morbidity [25–27].

**Fig. 3:**
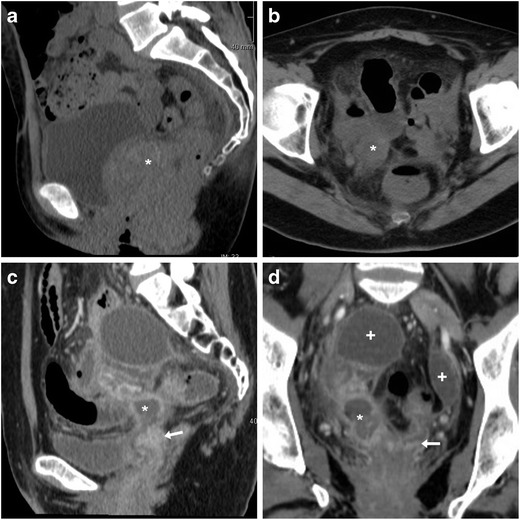
Two cases of vaginal vault haematoma. **A**) Typical example of midline haematoma after VH for GP in a 40-year-old female: precontrast sagittal CT image showing 5-cm hyperattenuating (45–50 Hounsfield units, HU) mass-like structure (*) above the vaginal dome, causing compression of the bladder. **B**–**D**) in a 35-year-old female, following VH for menorrhagia, a smaller (3 × 2 cm) paramedian vaginal vault haematoma (*) shows mild precontrast (**B**) hyperattenuation (40 HU), peripheral enhancement after iv contrast (**C**, **D**), abutting the upper right aspect of the vaginal dome (arrows). Both occurrences were successfully managed conservatively. Note: lymphocele (+). (Partially reproduced from open access ref. no [58])

At CT, median or paramedian haematomas generally measure 3 to 6 cm in size and are identified by their characteristic hyperattenuation. In CM-enhanced acquisition, the peripheral rim enhancement can be misleading as it may suggest an incorrect diagnosis of abscess (Fig. 3). However, in most cases, the gynaecologist is aware of this complication since it is easily detected clinically and sonographically [25–27].

### Lymphoceles

Lymphoceles are collections of lymphatic fluid which result from surgical injury to the lymphatic system. Strongly associated with lymph node dissection, uni- or bilateral lymphoceles are detected in up to 40% of patients after resection of gynaecologic tumours [4, 28] .

**Fig. 4:**
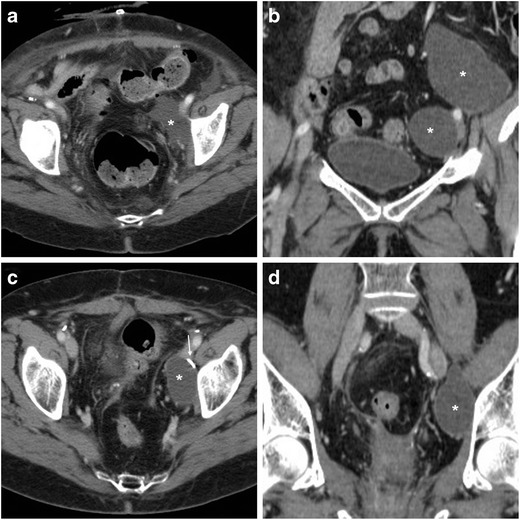
Usual CT appearance and short-time evolution of pelvic lymphoceles. **A**, **B**) Three days after AH for uterine body carcinoma, early CT (**A**) showed a fluid attenuation collection (*) without perceptible wall abutting the left external iliac vessels. Four weeks later, repeated CT (**B**) showed large left-sided lymphoceles (*) with unchanged homogeneous attenuation and development of a thin, regular wall. **C**, **D**) in another patient, early postoperative CT after radical AH showed a well-demarcated ovoid collection (*) with thin regular wall measuring 4 × 2.5 cm, adjacent to surgical clips (thin arrow in C) and left external iliac vessels

At CT, the characteristic appearance of a lymphocele is a laterally positioned pelvic collection with homogeneous fluid attenuation and signal intensity, generally adjacent to surgical clips and iliac blood vessels (Figs. 3 and 4). On early postoperative CT studies, lymphoceles lack a perceptible wall (Fig. 4a). Later on, lymphoceles progressively become round- or ovoid-shaped and better demarcated, and develop a thin regular contour which corresponds to the fibrotic wall without epithelial lining. Variably sized lymphoceles are generally asymptomatic and therefore incidentally found on cross-sectional imaging. The majority of cases tend to resolve spontaneously, may occasionally (20% of cases) persist 1 year after surgery and do not require treatment [28].

Some lymphoceles may cause ipsilateral lower limb oedema and pain, and may be treated by imaging-guided interventions. Percutaneous catheter drainage with and without sclerotherapy achieves technical success in 74.3% and 100% of cases, respectively. Recurrence occurs in up to 13% of patients and may require repeated drainage. Alternatively, open surgical or laparoscopic drainage and internal marsupialisation may be performed [29, 30].

**Fig. 5:**
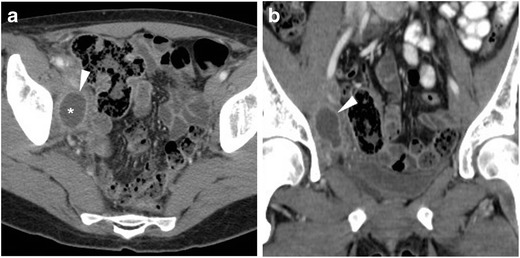
Delayed superinfection of a lymphocele 9 months after radical AH in a 41-year-old female, suffering from recurrent fever after chemotherapy. Contrast-enhanced CT (**A**, **B**) showed a fluid-like collection with thick, enhancing wall (arrowheads) in the right lateral pelvis, in the site of a small lymphocele described in a previous ultrasound (not shown). Signs of iliac-femoral thrombosis were absent. Fever, laboratory and imaging abnormalities ultimately regressed after intensive antibiotics (Partially reproduced with permission from ref. no [59])

Occasionally, complications such as deep venous thrombosis, bladder compression and superinfection may occur. The latter is heralded by fever, pelvic pain, leukocytosis and elevated acute phase reactants, and is suggested at imaging by enlargement of a known lymphocele, appearance of septations, internal inhomogeneity or by an abscess-like appearance with thickened enhancing walls (Fig. 5) [31].

### Surgical site infections

**Fig. 6:**
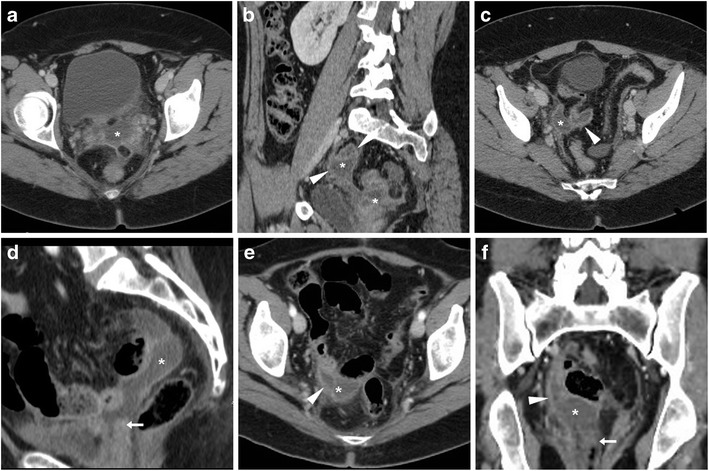
Multidetector CT appearances in two cases of surgical site infections. **A**–**C**) after recent laparoscopic hysterectomy (LH), clinical diagnosis of pelvic cellulitis corresponded to a bilobated fluid-like collection (*) with thin peripheral enhancement. **D**–**F**) after radical AH, a larger abscess (*) with peripheral enhancement (arrowheads) developed upwards from the normal-appearing vaginal dome (arrows) (Partially reproduced from open access ref. no [58])

Infections result from ascending polymicrobial contamination of the surgical site from the vagina and endocervix. Specific risk factors include patient features (such as obesity, diabetes, malnutrition, advanced ages, steroid use, history of radiation and bacterial vaginosis) and operation-related factors including prolonged duration, blood loss and staple closure. Among them, VC cellulitis represents superficial infection at the vaginal suture margin, and typically present with 5–10 postoperative days with fever, abdominal pain and palpation without masses. Conversely, deep infections encompass pelvic cellulitis and abscesses, often manifest after discharge from the hospital with pelvic pain, purulent vaginal secretions, marked oedema, tenderness and a mass at physical examination. CT helpfully complements physical examination by demonstrating the extent and topography of pelvic abscesses (Fig. 6), which may sometimes result from superinfection of a conservatively treated haematoma, lymphocele or urinoma. If clinically unresponsive to intensive antibiotics, abscesses require percutaneous or laparoscopic drainage [32].

Alternatively to infection, the presence of a fluid collection abutting the VC may occasionally correspond to VC dehiscence. Particularly in patients suffering from vaginal pain and bleeding, careful scrutiny of the VC for mural discontinuity and fat herniation is warranted to suggest to the gynaecologist the possibility of VC and request a focused physical examination [2, 9, 33].

The key CT differential diagnosis of a postoperative pelvic abscess is presence of oxidised regenerated cellulose (Surgicel®, Ethicon), a gauze-like bioabsorbable haemostatic material which mimics as a mixed gas-fluid collection and requires awareness of surgical details to avoid misinterpretation [34].

Finally, a peculiar condition underlying persistent postoperative fever is septic pelvic thrombophlebitis, which may result in systemic embolisation (to the lungs, brain and musculoskeletal system) if untreated [35, 36]. The CT findings include enlargement of hypogastric, iliac or femoral veins, with partial or complete luminal non-opacification, thickening and hyperenhancement of the vessel wall, and inflammatory stranding of the surrounding fat planes [9, 35, 37].

### Haemorrhage

After hysterectomy, bleeding is one of the most feared complications. Haemorrhage from surgical manipulation or inadequate vessel ligation may extend from the surgical site to the pelvis and abdominal cavity. Alternatively, during laparoscopic surgery, the abdominal wall, mesenteric or inferior epigastric vessels may be injured by insertion of Veress needles or trocars [23, 24].

**Fig. 7:**
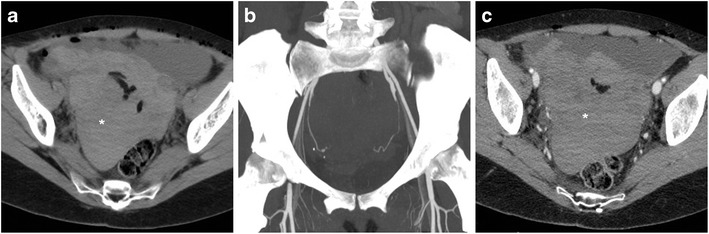
Large pelvic haematoma developing within 48 h after LH for uterine myoma, manifesting with hypotension and severe haemoglobin drop. Axial precontrast CT image (**A**) showed abundant, strongly hyperattenuating (60–70 HU) blood. CT angiography (maximum-intensity reformation **B**) and venous-phase CT acquisition (**C**) did not show active contrast medium extravasation suggesting active bleeding. Repeated laparoscopy was required for drainage of the peritoneal cavity and haemostasis

**Fig. 8:**
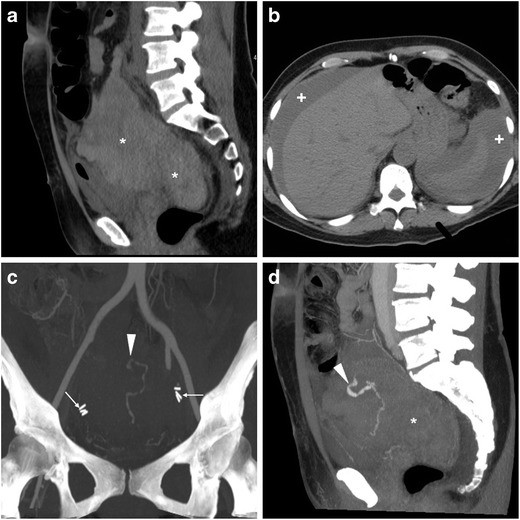
Haemoperitoneum after LH for uterine myomas, manifesting the next day with worsening pelvic pain and blood loss. Precontrast CT (**A**, **B**) images showed haemoperitoneum (+) in the upper abdomen, and a vast hyperattenuating haematoma (*) occupying most of the pelvis, causing compression on the urinary bladder. CT angiography (**C**) and venous-phase CT acquisition (**D**) showed active arterial bleeding as serpiginous contrast medium extravasation (arrowheads). Open surgery confirmed presence of nearly 1 l of blood in the abdominal cavity and arterial haemorrhage from the left-sided angle of the vaginal vault and small vessels of the ipsilateral ovarian pedicle, which was treated by coagulation and suture (Partially reproduced from open access ref. no. [60])

The use of CT is crucial to detect the presence, site, entity, age and features of iatrogenic haemorrhage. Haematomas are recognised by the characteristic 30–45-HU attenuation of fresh blood, which becomes even more hyperdense (HU > 60) after a few hours from clotting, and progressively develops inhomogeneity and fluid-fluid levels from mixing of clotted regions and serum from haemoglobin lysis. CT may reveal CM extravasation corresponding to active bleeding in either arterial or venous phase (Figs. 7 and 8) [7, 9].

If available, transarterial embolisation of the uterine or internal iliac artery is increasingly performed to control iatrogenic pelvic bleeding without repeated surgery [38].

### Bowel injury and obstruction

**Fig. 9:**
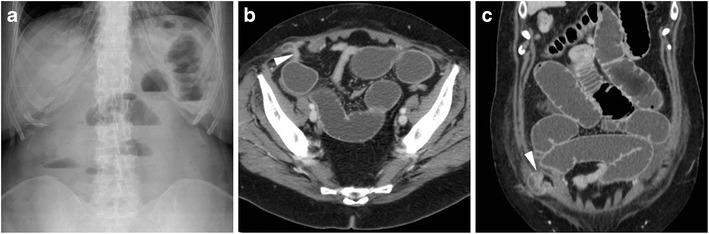
Small-bowel obstruction developing after recent LH. The patient was readmitted with plain radiographic (**A**) evidence of distended bowel loops with multiple fluid-fluid levels. CT (**B**, **C**) confirmed distended jejunal and ileal loops with abundant intraluminal fluid, caused by herniation (arrowhead) into a laparoscopic trocar access site

Compared to postoperative ileus, bowel obstruction is relatively rare. Within and immediately after the postoperative hospitalisation, the most frequent cause is represented by incision and port site (Fig. 9) hernias developing respectively after open and laparoscopic surgery. Risk factors include advanced age, obesity, duration of surgery, use of large-bore trocars and ports for specimen removal. Due to the small size of the defect, both sites have a high likelihood of causing obstruction. Borrowing from experience with spontaneous acute abdomens, CT effectively triages presence and degree of obstruction, transition point (Fig. 9) and possible signs of strangulation [22–24].

**Fig. 10:**
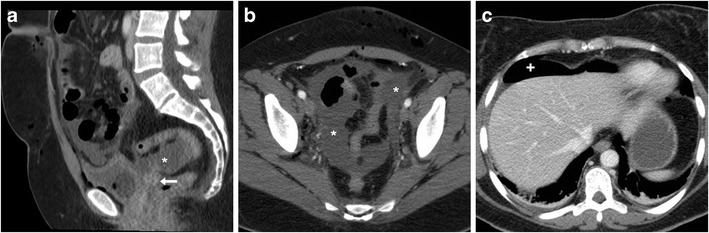
Peritonitis developing 4 days after LH. Contrast-enhanced CT images showed normal findings at the vaginal dome (arrow in **A**), moderate pelvic effusion (*) with thin serosal enhancement, and residual intraperitoneal air (+). Laparotomy identified a focal ileal perforation which was repaired

**Fig. 11:**
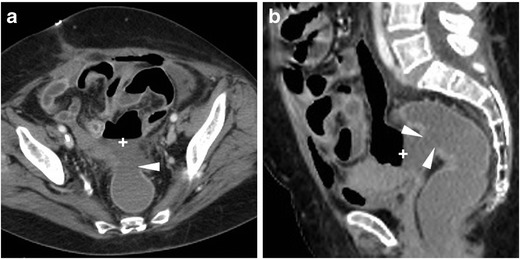
Large-bowel perforation following AH. Post-surgical CT showed a distended rectum and sigmoid, and a clear-cut mural discontinuity (arrowheads) of the anterior aspect of the distal sigmoid colon, from which fluid and air flowed in the peritoneal cavity. Surgical reoperation was necessary

Although very uncommon, iatrogenic intestinal injuries represent potentially serious complications, especially if unrecognised at the time of surgery. Delayed diagnosis corresponds to high risk of perforation, faecal peritonitis, sepsis and death. After both AH and VH, most injuries involve the rectum and sigmoid colon. Conversely, albeit incidence is similar, laparoscopic surgery may damage the small bowel during Veress needle or trocar insertion. Radiologists should suggest possible bowel injury when faced with abundant, persistent or increasing pneumoperitoneum, with peritonitis with effusion and enhancing serosa (Fig. 10), or with unexplained abscess collections. The rare but more specific signs include identification of focal bowel wall thickening, discontinuity (Fig. 11) or non-enhancement. Unfortunately, CT sensitivity is approximately 70%, and negative CT findings despite serious bowel complications have been reported [9, 39].

## Post-hysterectomy urologic complications

Despite technical advancements and surgeons’ experience, due to the close anatomic relationship between the urinary and female genital tracts, urologic injuries represent typical complications of gynaecologic surgery and a potential cause of litigation. According to the European Association of Urology (EAU) guidelines, early recognition facilitates immediate repair, improves patients’ outcome and decreases the likelihood of long-term sequelae such as renal function impairment [8, 40].

General risk factors for urologic complications include obesity, diabetes, radical hysterectomy including lymph node dissection for malignancy, adhesions from previous surgeries and history of irradiation. In descending order of frequency, urologic injuries affect the urinary bladder (60–70% of cases), the ureter (24–30%) and the vagina (2.1%). Acute urologic injuries manifest during the first postoperative days with a combination of pelvic or flank pain, abdominal distension, ileus, peritonitis, fever, decreased urinary output, worsening renal function, haematuria and abnormal urinalysis [41–48].

### Ureteral injuries

Mechanisms of iatrogenic damage to the ureters include direct manipulation, inadvertent ligation or kinking with a suture, thermal injury and ischaemia from devascularisation. In patients with distorted pelvic anatomy, prophylactic stenting is beneficial to decrease risks. Unfortunately, ureteral damage is unrecognised during hysterectomy in almost two thirds of patients and delayed diagnosis (weeks to months after surgery) with hydronephrosis and permanent loss of renal function is not unusual [8, 43, 44, 49, 50].

**Fig. 12:**
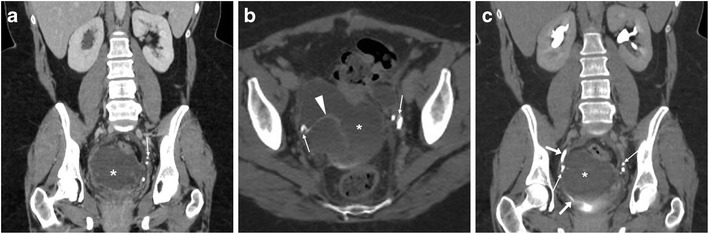
Ureteral injury manifesting with fever and pelvic tenderness 3 days after TLH. Vaginal examination and sonography (not shown) detected a sizeable hypo-anechoic pelvic collection, initially interpreted as a vaginal vault haematoma, which rapidly increased in volume. CT confirmed a large fluid-attenuation collection (*) cranial to the urinary bladder and vagina, which in the excretory phase (**B**, **C**) was partially opacified by enhanced urine (arrowhead in **B**) leaking from the right ureter. The latter was opacified (arrow in C) distally to the injury, consistent with an incomplete laceration. Note surgical clips (thin arrows). Conservative treatment included ureteral stenting and prolonged catheterisation. A month later, follow-up CT (not shown) showed near-complete resolution of the urinoma (Partially reproduced with permission from ref. no [61])

**Fig. 13:**
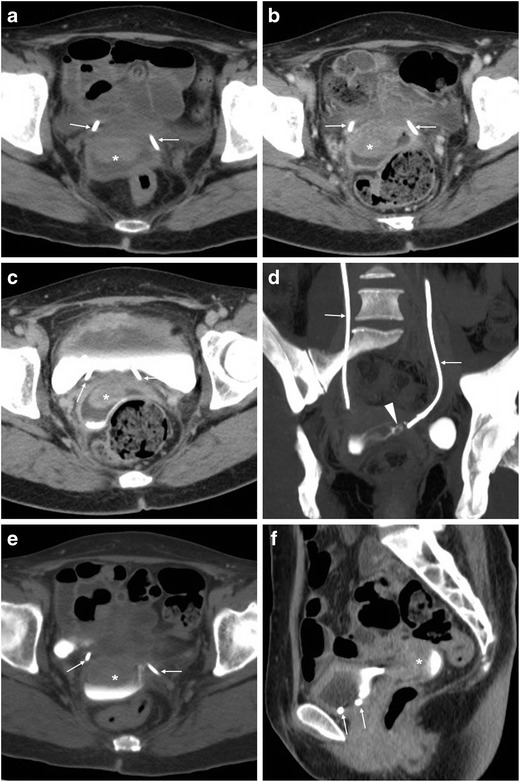
Another case of ureteral injury shortly after LH. Despite preoperative bilateral ureteral stenting (thin arrows), unenhanced CT images (**A**) showed a mixed-fluid and hyperattenuating pelvic collection (*), with corresponding peripheral enhancement in the venous phase (**B**) and filling by opacified urine in the excretory phase (**C**). On additional delayed acquisition, leakage from the most distal tract of the left ureter (arrowheads in **D**, **E**) and progressive opacification of the urinoma (*) were seen. Endourological treatment was performed (Partially reproduced from open access ref. no [58])

According to the EAU guidelines, appropriate therapeutic planning requires thorough assessment of the site, features and degree of ureteral injuries using excretory-phase CT [8]. The right and left ureters are injured with similar frequency. The typical (80–90% of cases) site of injury is the lower ureteral third, either at the pelvic brim or along the pelvic sidewall at uterine artery crossing. The main CT pattern of ureteral injury is partial or complete disruption, which is heralded by extravasation of urine (Figs. 12 and 13). Following recent hysterectomy, fluid-attenuation (0–20 HU) collections abutting the ureters should be viewed with suspect. The diagnosis of urinoma is confirmed by enhancement on excretory-phase scanning, with inhomogeneous opacification (denser close to the leaking source) which progresses on repeated delayed scanning. Preserved opacification of the ureteral segment distal to the leaking site allows differentiating partial lacerations from complete disruption [7, 9, 51–53].

**Fig. 14:**
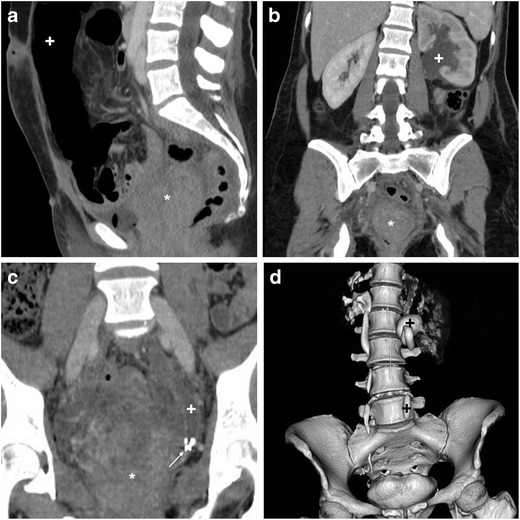
Ligation of the left ureter manifesting 24 h after AH. Unenhanced CT (**A**) showed a sizeable vaginal vault haematoma (*) and residual pneumoperitoneum (+) as expected findings. After intravenous contrast, the left kidney showed hydronephrosis (+) and impaired nephrogram compared to contralateral side, caused by abrupt stricture of the distal ureter (+ in **C**) in the site of surgical clips (thin arrow in **C**). Surgical reoperation including clip removal was required to manage the obstruction

**Fig. 15:**
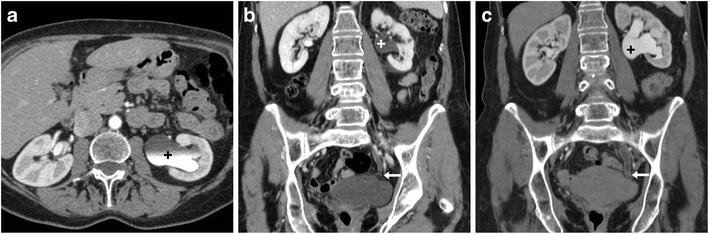
Long-term effects of an unrecognised ureteral stricture following AH 1 year earlier. Follow-up CT with biphasic contrast injection showed left-sided hydronephrosis (+) from tight ureteral stricture (arrow in **C**) which required endourological management

The second pattern of ureteral injury is ligation without urine extravasation, which often occurs at the site of surgical clips, causes upstream hydronephrosis and may be either acute (Fig. 14) or asymptomatic (Fig. 15). Nowadays, percutaneous nephrostomy, antegrade recanalisation by endourology techniques and long-term stent placement are the preferred management for both ureteral obstruction and laceration. Alternatively, open or laparoscopic surgical repair with suturing, end-to-end uretero-ureterostomies or uretero-neocystostomy may be necessary [49, 50, 54].

### Bladder injury and fistulas

The urinary bladder may be damaged during surgical dissection or from laparoscopic trocar insertion. Most bladder injuries are recognised intraoperatively using dyes and cystoscopy, and immediately repaired [44, 55].

**Fig. 16:**
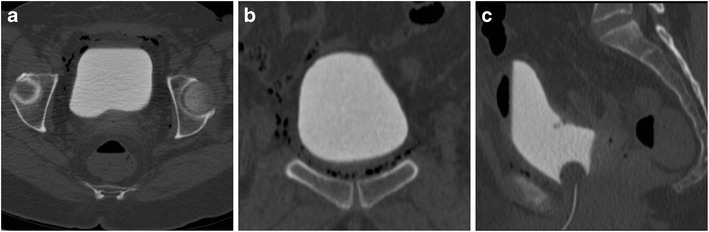
Multidetector CT-cystography performed 4 h after radical surgery for ovarian cystoadenocarcinoma, with intraoperative suspicion of bladder rupture. Multiplanar images show optimal distension of the normal-shaped urinary bladder by diluted contrast medium instilled via the Foley catheter. Note perivesical gas bubbles. The lack of extravasated contrast in the perivesical spaces allowed confident exclusion of either intra- or extraperitoneal bladder injury and vesico-vaginal fistula (Reproduced from open access ref. no. [18])

After surgery, the CT hallmark of bladder injury is represented by either extraperitoneal or intraperitoneal urine extravasation at excretory-phase CT. Since a Foley catheter is generally in place shortly after hysterectomy, minor bladder injuries and suspected urinomas should be investigated using CT cystography which confirms bladder wall integrity, ruling out possible leakages, when retrograde filling with at least 250 ml of diluted contrast is obtained (Fig. 16) [7, 9, 18]. Whereas extraperitoneal leakage is treated conservatively with bladder drainage, intraperitoneal injuries require surgical exploration and repair [8].

Alternatively, hysterectomy may result in formation of a vesico-vaginal fistula (VVF), which accounts for over two thirds of iatrogenic pelvic fistulas. The risk is highest (0.74%) during radical AH secondary to extensive parametrial and nodal dissection. The typical manifestations include continuous urinary leakage, foul odours or discharge from the vagina. Clinical diagnosis of VVF is often challenging, and requires a combination of vaginal examination, cystoscopy, bladder filling with dye and biochemical assay of discharge fluid for high creatinine levels [42, 44, 47, 56].

**Fig. 17:**
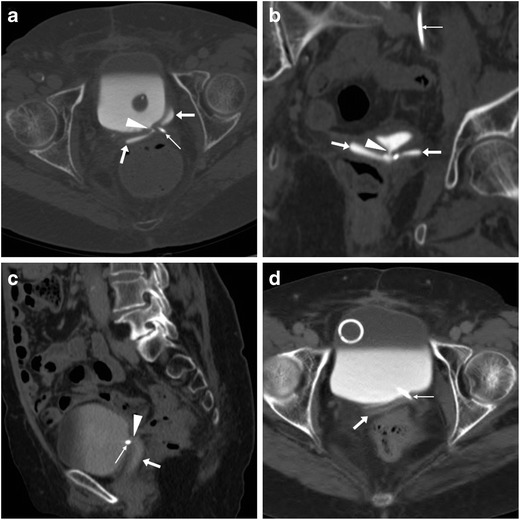
Vesico-vaginal fistula (VVF) developing after radical AH and radiotherapy for endometrial carcinoma in a 66-year-old female, manifesting with vaginal discharge without abnormal findings at gynaecological examination. Excretory-phase CT (**A**, **B**) showed a well- opacified urinary bladder with a Foley catheter and left-sided ureteral stent (thin arrow) in place, lateral retraction and opacification of the vagina (arrows) through a short communication (arrowheads) consistent with VVF. After vaginal electrocoagulation, long-term antibiotics and ureteral stenting, repeated CT urography 6 months later (**C**) showed persistent vaginal opacification (arrow) through the high-output supratrigonal VVF. A year later, CT follow-up (**D**) showed non-opacified vagina (arrow) indicating fistula closure (Partially reproduced with permission from ref. no. [62])

At imaging, a VVF is suggested by identification of air and/or fluid in the vaginal lumen. Excretory-phase CT or CT cystography are useful for treatment planning as they can confirm and directly visualise the opacified abnormal track (Fig. 17) [7, 9, 18, 57]. Albeit some VVF seal during with prolonged catheterisation, most cases ultimately require surgical repair [42, 44, 47, 56].

## Conclusion

Imaging plays an increasingly pivotal role in the diagnosis of postoperative complications following hysterectomy, such as infections, haemorrhage, bowel perforation or obstruction, urologic injuries and fistulas. In our experience, multidetector CT allows a comprehensive assessment of the operated pelvis, and is therefore recommended to elucidate suspected post-hysterectomy complications and to provide a consistent basis for choosing between conservative, percutaneous or open surgical management.
